# Effective Antioxidants as Plausible Ligands in Chromium(III) Supplementation: How Complexation Modulates Catechol-Based Polyphenols

**DOI:** 10.3390/molecules30224467

**Published:** 2025-11-19

**Authors:** Hanna Lewandowska, Zhe Chen, Krystian Marszałek, Włodzimierz Lewandowski, Renata Świsłocka

**Affiliations:** 1 School of Health & Medical Sciences, Vizja University in Warsaw, Okopowa 59, 01-043 Warsaw, Poland; 2 Centre for Radiation Research and Technology, Institute of Nuclear Chemistry and Technology, 16 Dorodna St., 03-195 Warsaw, Poland; 3 Department of Fruit and Vegetable Product Technology, Prof. Wacław Dąbrowski Institute of Agricultural and Food Biotechnology—State Research Institute, Rakowiecka 36, 02-532 Warsaw, Polandkrystian.marszalek@ibprs.pl (K.M.); 4 Department of Chemistry Biology and Biotechnology, Bialystok University of Technology, Wiejska 45E, 15-351 Bialystok, Polandr.swislocka@pb.edu.pl (R.Ś.)

**Keywords:** effective antioxidants, polyphenols, caffeic acid, 3,4-dihydroxybenzoic acid, metal complexes, chromium(III), antidiabetic

## Abstract

This study examines the impact of metal coordination on the antioxidant and pro-oxidant properties of 3,4-dihydroxybenzoic acid (3,4-DHBA) and caffeic acid (CA). Their Na(I), K(I) salts and Cr(III) complexes were evaluated in vitro using radical scavenging assays (ABTS, DPPH, hydroxyl, and superoxide), ferric- and cupric-reducing power, and inhibition of linoleic acid peroxidation. Alkali metal coordination generally decreased radical scavenging activity, though K complexes and Cr–3,4-DHBA improved lipid peroxidation inhibition. Cr(III) chelation produced ligand-dependent effects: it markedly increased the reducing power of CA while reducing that of 3,4-DHBA and uniquely promoted pro-oxidant behavior in CA under superoxide conditions. These outcomes reflect how chromium chelation alters electronic distribution and charge transfer, enhancing reducing power in single-electron transfer assays while enabling redox cycling in radical scavenging systems, underscoring its dual and ligand-dependent biological significance.

## 1. Introduction

Aromatic chromium–carboxylate complexes, investigated since the 1980s for their metabolic and antioxidant potential, are considered important supplemental forms in the context of diabetes [1]. In the current scientific literature, the role of chromium in biological processes remains an open and highly debated issue [1,2]. Findings from both clinical and experimental studies are inconsistent—some report potentially beneficial effects of Cr(III) compounds on carbohydrate and lipid metabolism, while others do not confirm any clear outcomes. As a result, the mechanisms of chromium’s action in living systems are largely unknown and unexplained. It is unclear whether the observed effects arise from direct involvement of Cr(III) in metabolic pathways, from its ability to modulate oxidative stress through complex formation with ligands, or from indirect influences on the bioavailability of other micronutrients. The current state of knowledge allows us to conclude that chromium interacts with biological systems in a complex manner, dependent on its chemical form, dosage, exposure time, and molecular environment; however, no coherent and widely accepted theory exists to explain its mechanism of action [3]. Chromium picolinate and chromium nicotinate are two widely used forms of trivalent chromium in dietary supplements [2]. Both are marketed for their beneficial roles in glucose metabolism, particularly for improving insulin sensitivity and glycemic control [1]. Indeed, numerous studies indicate that supplemental chromium can potentiate insulin’s activity in insulin-resistant states. For example, clinical research has shown that chromium picolinate supplementation can reduce insulin resistance and improve markers of glycemic control [1,3,4]. The insulin-sensitizing effect of trivalent chromium was attributed to its action at the cellular level on insulin signaling pathways [5]. Beyond their influence on insulin and glucose homeostasis, chromium picolinate and nicotinate have been reported to confer indirect antioxidant benefits as a result of improved metabolic control [6]. In diabetic animal models, chromium supplementation (particularly in the form of picolinate and nicotinate) has been reported to enhance antioxidant enzymes, lower lipid peroxidation markers, and reduce pro-inflammatory cytokines and cholesterol levels. However, these findings remain disputed [2,7,8].

It is important to emphasize that, despite observed reductions in oxidative stress markers in some models, chromium picolinate and nicotinate are not regarded as direct antioxidants, since their ligands—picolinic and nicotinic acids—lack intrinsic redox activity [8]. In contrast, phenolic acids such as caffeic acid and 3,4-dihydroxybenzoic acid provide a stronger biological rationale. Belonging to the catechol family (throughout this manuscript, we use the term “catechol moiety” to refer specifically to the adjacent dihydroxy group and “catechol family” to refer more broadly to compounds (such as CA and 3,4-DHBA) that contain this structural motif), they possess adjacent hydroxyl groups that are potent electron donors and form stable complexes with trivalent chromium. These ligands not only contribute to complex stability and bioavailability but also bring inherent antioxidant and anti-inflammatory properties [9,10]. When complexed with chromium, catecholic ligands could therefore combine chromium’s regulatory and insulin-sensitizing functions with direct radical scavenging activity, offering a dual biological effect.

This concept fits within a broader field of research showing that the complexation of antioxidants with metal ions of high ionic potential enhances electron delocalization, stabilizes radical intermediates, and improves overall antioxidant efficiency [9,10]. Metal–polyphenol complexes are emerging as a dynamic area in food chemistry, functional nutrition, and pharmaceutical sciences, with potential applications ranging from disease prevention to food preservation [11]. Within this framework, 3,4-dihydroxybenzoic acid (3,4-DHBA, protocatechuic acid) and caffeic acid are of particular interest, as they are naturally abundant, well-characterized plant phenolics with established radical scavenging and health-promoting properties [12,13,14,15].

3,4-Dihydroxybenzoic acid and caffeic acid are catechol-based phenolic acids widely present in fruits, vegetables, and medicinal plants. Both are effective antioxidants because the catechol group provides strong electron-donating and radical-stabilizing ability. Their activity, however, differs due to structural features: hydroxycinnamic acids such as caffeic acid possess a conjugated –CH=CH–COOH side chain that enhances electron delocalization and generally confers higher radical scavenging potency than hydroxybenzoic acids like 3,4-DHBA, which lack this conjugation [16,17,18,19,20,21,22,23]. Additional substituents such as methoxy groups further modulate activity by altering charge distribution and stabilizing phenoxyl radicals. Thus, the distinct structural frameworks of caffeic acid and 3,4-DHBA provide a rationale for comparing their behavior upon metal coordination [16,17,18,19,20,21,22,23].

In recent years, metal coordination has emerged as a strategy to enhance the biological activity of polyphenolic compounds, including their antioxidant properties. Many studies have reported that coordination with metal ions can significantly alter the chemical and biological characteristics of the original organic compound, affecting its solubility, stability, redox potential, and ability to scavenge ROS [24,25,26]. For instance, Mei et al. (2012) have shown that a complex of Zn(II) with curcumin offers greater protection against ethanol-induced gastric mucosa injury than curcumin alone, demonstrating the therapeutic potential of such complexes [27]. The complexation of rutin and galangin with metals like Cu(II), Fe(II), Al(III), and Zn(II) has significantly improved their DPPH radical scavenging activity, as shown by the lower Inhibitory Concentration 50% (IC_50_) values of the complexes in comparison to the free polyphenols [28]. A complex of gentisic acid with Ca(II) has demonstrated enhanced antioxidant activity in various assays, including DPPH, Ferric-Reducing Antioxidant Power (FRAP), and Cupric Reducing Antioxidant Capacity (CUPRAC), compared to gentisic acid alone [29]. Metal complexation enhances the inherent antioxidant activity of phenolic compounds and often improves their bioavailability. For instance, the bioavailability of quercetin increases significantly when complexed with Cu(II) ions [30]. Furthermore, the chelation of metal ions with phenolic compounds can affect the kinetics of the antioxidant reactions, typically accelerating them and thereby leading to more efficient radical scavenging [31]. The enhanced antiradical properties of metal complexes are primarily due to the addition of radical scavenging metal centers, which provide new sites for radical capture that are absent in the free ligands [28].

Both CA and 3,4-DHBA can chelate metals via their phenolic and carboxylate groups. Complexing these antioxidants with metals might stabilize otherwise reactive ions and deliver them safely in biological systems. Caffeic acid can form stable complexes with numerous metals (Fe^2+^/Fe^3+^, Cu^2+^, Al^3+^, Eu^3+^, Mn^2+^, and others). Its dianionic and trianionic forms offer different binding modes (bidentate coordination via catechol oxygens and/or via the carboxylate), leading to high stability constants in aqueous solution [14,32,33].

Complexation alters the electron density on the phenolic oxygen atoms and may either inhibit or enhance antioxidant activity depending on the metal and the type of assay. Studies on CA show that catechol hydroxyl groups’ chelation mode modulates oxidative chemistry. Density-functional calculations on Fe(II)/CA complexes showed that the anionic forms of CA “trap Fe(II)” and make thermodynamically stable complexes. Upon this complexation, hydrogen peroxide reacts much less readily with the metal center, suppressing Fenton reaction. Oxidation of Fe(II) to Fe(III) in the presence of CA becomes less favorable, and CA effectively “deactivates hydroxyl radical formation by sequestering the ferrous ion” [34]. Radical scavenging decreases when coordination occurs through the catechol moiety. Lanthanide (Eu, Gd, Dy) complexes of caffeic acid exhibited higher IC_50_ values and lower FRAP values than the free ligand [35]. Coordination through the catechol withdraws electron density, reducing the ability to donate hydrogen atoms and limiting resonance stabilization of the resulting phenoxyl radicals. Eu(III)–caffeic acid complexes similarly showed lower DPPH and ABTS scavenging than caffeic acid [14]. The decreased activity contrasts with p-coumaric acid complexes, where the carboxylate binds and leaves the phenolic hydroxyls free [35], highlighting the importance of the binding site.

Caffeic acid prevents hydroxyl radical formation in Fenton systems by forming Fe–CA complexes [36]. The complex competes with water in the iron coordination sphere, making the metal less available to participate in radical-generating reactions. At higher pH levels, deprotonation enhances the strength of complexation and improves antioxidant activity. Similar chelation prevents LDL oxidation in the presence of Cu^2+^ [37]. Thus, in systems where radical generation depends on metal catalysis, chelation by caffeic acid enhances antioxidant protection. In Fe(III) and Cu(II) complexes of chlorogenic acid, radical scavenging capacity decreased, but lipid peroxidation inhibition increased [38]. This suggests that metal–phenol complexes may act as chain-breaking antioxidants in hydrophobic environments rather than as direct radical scavengers. Complexation could improve solubility in lipid membranes, enabling the complex to intercept lipid-derived radicals more effectively.

Fewer studies have examined 3,4-DHBA complexes with metals. Potentiometric measurements show that 3,4-DHBA forms Cu^2+^ complexes similar in stability to those of hydrocaffeic acid (3,4-dihydroxyhydrocinnamic acid), indicating that the catechol moiety is critical for binding [39]. A theoretical DFT study on protocatechuic aldehyde predicts exergonic formation of Fe^2+^/Fe^3+^–3,4-DHBA complexes; the most stable complex has a 1:3 ratio and strongly suppresses Fenton chemistry [40]. In environmental chemistry, 3,4-DHBA complexes with Fe^3+^ accelerate the Fe^3+^/Fe^2+^ cycle and boost hydroxyl radical generation in Fenton processes, demonstrating that 3,4-DHBA can act as a pro-oxidant under certain conditions [41].

Caffeic acid and 3,4-dihydroxybenzoic acid (3,4-DHBA) were deliberately selected as model compounds. They are recognized as effective antioxidants due to their catechol moieties, which confer strong radical scavenging and redox activity. Both ligands share the same number and position of hydroxyl groups on the aromatic ring but differ in the length of the conjugated double-bond system. This creates the opportunity to confirm whether extending the conjugated system enhances the antioxidant properties of the ligand. In this context, the effect of metal complexation is also of particular interest. The rationale of our study was to explore whether these established antioxidants could serve as potential ligands for chromium(III) supplementation, with the idea that coordinating Cr(III) with potent antioxidants might not only stabilize the metal ion but also mitigate safety concerns by coupling chromium with protective antioxidant properties. To address this, we systematically investigated how the interaction of caffeic acid and 3,4-DHBA with Na(I), K(I), and Cr(III) influences their antioxidant and pro-oxidant behavior, thereby distinguishing the effects of simple salt formation from those of true metal complexation. Using a broad panel of in vitro assays (ABTS, DPPH, hydroxyl radical, superoxide, FRAP, CUPRAC, pro-oxidant activity, and linoleic acid peroxidation inhibition), we evaluated how metal binding modulates the functional behavior of these catecholate salts. The results provide mechanistic insight into the biological activity of catecholates, with particular emphasis on chromium complexes, which are of current interest in the ongoing debate on chromium’s antioxidant effects and its potential role in diabetes.

## 2. Results

### 2.1. FTIR Identification of the Obtained Complexes and Ligand Stoichiometry

The spectra of all the compounds were recorded and are presented in Supplementary Figures S1 and S2. The vibration bands of the aromatic system were numbered according to Varsanyi’s classification [42]. The 3,4-DHBA spectra exhibit a characteristic band originating from vibrations of the carbonyl group: stretching vibrations νC=O occurring at 1675 cm^−1^, in-plane bending vibrations βC=O located at 799 cm^−1^, and out-of-plane bending γ(C=O) at 636 cm^−1^. The disappearance of these bands in the spectra of salts and complexes indicates the participation of the carboxyl group in metal coordination. At the same time, bands corresponding to carboxylate anion vibrations appear, further supporting the coordination process. These are the bands of the asymmetric stretching vibrations ν_as_COO^−^ located in the range 1519–1481 cm^−1^ and the symmetric stretching vibrations ν_s_COO^−^ in the range 1410–1375 cm^−1^. Furthermore, the spectra contain bands associated with in-plane bending vibrations β_s_COO^−^ (symmetric) in the range 977–974 cm^−1^ and β_as_COO^−^ (asymmetric) in the range 603–592 cm^−1^ as well as out-of-plane bending (γ_s_COO−) lying in the range 717–707 cm^−1^ (see Table 1 and Table 2, Supplementary Figures S1 and S2).

The spectra of 3,4-DHBA and its salts and complexes also exhibit characteristic bands originating from vibrations of hydroxyl groups attached to the aromatic ring in the range 3476–3314 cm^−1^. In addition, the spectra contain characteristic bands originating from stretching vibrations of the aromatic ring, i.e., 8a, 8b, 19a, 19b, and 14.

Deprotonation of the carboxyl group in caffeic acid, followed by metal salt formation, induced characteristic changes in the IR spectra of the Cr(III)–caffeate complexes compared with those of the free ligand. Disappearance of valence vibration bands can be observed for stretching ν(C=O) at 1645 cm^−1^; deformation vibrations β(C=O) at 815 cm^−1^; as well as γ(C=O) at 698 cm^−1^ of the carbonyl group; disappearance of stretching vibrations ν(OH) in the range of 2837–2574 cm^−1^; appearance of bands of asymmetric and symmetric vibrations of the carboxylate anion ν_as_(COO^−^) in the range of 1526–1490 cm^−1^, ν_s_(COO^−^) in the range of 1410–1375 cm^−1^, β_s_(COO^−^) in the range of 977–974 cm^−1^, β_as_(COO^−^) in the range of 602–592 cm^−1^, and γ_s_(COO^−^) in the range of 717–707 cm^−1^; and disappearance or changes in positions and intensities of some aromatic bands.

Based on the criteria of Nakamoto and McCarthy, which consider the separation between the asymmetric and symmetric stretching vibrations of the carboxylate group, the coordination mode of the COO^−^ group can be predicted [43]. For the Cr(III)–caffeic acid complex, Δν(COO^−^) is much smaller than in the sodium salt (ΔνCr = 80 cm^−1^ vs. ΔνNa = 144 cm^−1^), consistent with bidentate chelation of the carboxylate group (Table 1).

**Table 1:** The wavenumbers (cm^−1^), intensities, and assignments of bands observed in the experimental FTIR spectra of CA and Na, K, and Cr(III)-caffeate.

CA [44]	Caffeates			Assignment (Varsanyi’s Designation)
	Na	K	Cr(III)	
3431 s ^a^	3415 m	3415 vs	3415 s	ν ^b^(OH)_ar_
3231 s	3227 m	3234 m	3233 m	ν(OH)_ar_
3027 w	3034 vw	3025 vw	3026 w	ν(CH) (2), ν(CH)_C=C_
2988 w	2981 vw			ν(CH) (20b), ν(CH)_C=C_
2926 w				ν(CH) (20a)
2837–2573 m				ν(OH)
1645 vs				ν(C=O)
1620 vs	1638 m	1638 s	1638 s	ν(C=C)
1599 s	1600 m	1616 vs	1619 vs	ν(CC) (8a)
1530 m		1553 m		ν(CC) (8b)
-	1519 vs	1526 s	1490 s	ν_as_(COO^−^)
1524 m	1480 w			ν(CC) (19a)
1450 vs	1417 s	1405 s	1447 m	ν(CC) (19b)
	1375 vs	1378 s	1410 vs	ν_s_(COO^−^)
1352 m	1308 m		1325 sh	ν(CC) (14), β(OH)_ar_, β(CH)_C=C_
	1293 s	1297 s		β(CH) (3), β(CH)_C=C_
1286 s				β(OH)
1279 vs	1280 vs	1276 s	1276 vs	ν(C-OH)
	1270 s			β(CH)_C=C_
1217 s	1208 m	1221 m	1219 m	β(OH)
1175 m	1173 m	1194 m	1174 m	β(CH) (18a)
1121 s	1116 s	1113 m	1117 s	β(CH) (18b)
-	976 m	977 m	974 m	β_s_(COO^−^)
974 m				γ(CH) (17b)
935 w	918 w	921 vw	936 w	γ(CH) (17a)
849 m	851 m	851 m	851 m	γ(CH) (5), γ(CH)_C=C_
815 m	-			β(C=O)
800 m	808 m	800 m	817 m	γ(CH) (10a)
779 m	775 m	780 sh	780 w	α(CCC) (12)
-	712 m	707 m	717 w	γ_s_(COO^−^)
698 m	-			γ(C=O)
648 m	-			β(C=O)
			626 m	φ(CC) (16a)
602 w	610 m	622 m		α(CCC) (6a)
-	595 w	592 sh	602 m	β_as_(COO^−^)
552 w	566 m	537 w	551 w	α(CCC) (6b)
	515 w			γ(OH)_ar_
457 w	450 w	478 w	457 w	φ(CC) (16b)

^a^ s—strong; m—medium; w—weak; v—very; sh—shoulder; ^b^ the symbol “ν” denotes stretching vibrations; “β” denotes in-plane bending modes; “γ” designates out-of-plane bending modes; “φ(CCC)” denotes the aromatic ring out-of-plane bending modes and “α(CCC)” designates the aromatic ring in-plane bending modes.

**Table 2:** The wavenumbers (cm^−1^), intensities, and assignments of the bands observed in the experimental FTIR spectra of 3,4-DHBA, its Na, K, salts, and Cr(III) complex.

3,4-DHBA	3,4-DHBA Complexes			Assignment (Varsanyi’s Designation)
	Na	K	Cr(III)	
3475 s	3473 m	3476 vs	3472 s	ν(OH)_ar_
3414 vs	3414 s	3415 vs	3415 vs	ν(OH)_ar_
3233 m	3240 vw	3234 w	3212 w	ν(CH) (2)
3080 m	2923 vw			ν(CH) (20b)
2950–2559 m				ν(OH)
1675 vs				ν(C=O)
1617 s	1616 m	1617 vs	1628 sh	ν(CC) (8a)
1600 s		1579 m	1617 m	ν(CC) (8b)
	1545 s	1560 s	1580 m	ν_as_(COO^−^)
1529 m	1524 s	1522 m	1534 sh	ν(CC) (19b)
1467 m	1491 sh		1497 m	ν(CC) (19a)
	1429 s	1426 m	1426 s	
1420 s				β(OH)
	1391 vs	1396 vs	1388 vs	ν_s_(COO^−^)
1382 m				β(OH)
	1361 s	1366 m		ν(CC) (14)
1300 vs	1294 vs	1283 vs	1274 vs	ν(C-OH)
1252 s	1245 m	1231 m		β(OH)
1175 m	1209 m	1171 m	1215 m	β(CH) (18a)
1129 m	1133 m	1136 m	1127 m	ν(CH) (13)
1096 m	1096 s	1096 m	1104 m	β(CH) (18b)
942 m	952 m	950 m	953 m	γ(CH) (7b)
	879 w	886 m	888 w	β_s_(COO^−^)
849 m	834 sh	837 w	823 m	γ(CH) (5)
823 m		811 w	799 m	γ(CH) (11)
799 w	-			β(C=O)
	811 m	789 m		γ_s_(COO^−^)
763 s	775 s	773 s	782 m	α(CCC) (12)
	649 m	644 m	661 m	γ(OH)
636 m	-			γ(C=O)
	610 w	623 sh	630 w	β_as_(COO^−^)
558 m	547 w	539 w	517 m	α(CCC) (6a)
449 w	447 vw	445 vw		α(CCC) (6b)

s—strong; m—medium; w—weak; v—very; sh—shoulder; the symbol “ν” denotes stretching vibrations; “β” denotes in-plane bending modes; “γ” designates out-of-plane bending modes; “φ(CCC)” denotes the aromatic ring out-of-plane bending modes and “α(CCC)” designates the aromatic ring in-plane bending modes.

In contrast, for the Cr(III)–3,4-dihydroxybenzoic acid complex, the Δν value is larger than in the sodium salt (ΔνCr = 192 cm^−1^ vs. ΔνNa = 154 cm^−1^), indicating that the carboxylate coordinates monodentately or interacts primarily in an ionic manner.

In summary, the Δν(COO^−^) analysis demonstrates that coordination in both chromium complexes occurs primarily through the deprotonated carboxylate groups. The ν(OH) stretching region appears in the range characteristic of protonated aromatic hydroxyl groups, and its broadening is consistent with hydrogen-bonding interactions, likely involving water molecules associated with the complexes.

### 2.2. Complex Stoichiometry from Elemental and Ash Analysis

**Ash content:** For the CA–Cr complex, the ash content was 12.97% ± 0.02, which corresponds to a chromium content of about 8.87%. From these values, the calculated number of ligands per chromium atom was 2.98. For the PACr complex, the ash content was 15.30% ± 0.45, corresponding to a chromium content of about 10.45%. The ligand-to-chromium ratio was calculated as 2.91.

**Elemental analysis:** The experimental elemental analysis yielded the following results: for CA–Cr, C = 43.04%, H = 5.16%; for PACr, C = 44.06%, H = 3.86%. From the analysis of the ash content and elemental analysis, the best fit for these data corresponds to the formulations Cr(C_9_H_7_O_4_)_3_·9H_2_O for CA–Cr and Cr(C_7_H_5_O_4_)_3_·3H_2_O for 3,4-DHBA-Cr. The calculated and experimental elemental compositions show close agreement, as presented in Table 3.

The relatively high water content inferred from these formulations is consistent with the broad O–H stretching region in the FTIR spectra.

Elemental analysis and FTIR data together indicate that coordination in both chromium complexes occurs through the deprotonated carboxylate groups. No reasonable fit to the analytical data was obtained when assuming deprotonated catechol groups, confirming the absence of counter-ions and supporting the formation of neutral tris(carboxylato)chromium(III) complexes.

### 2.3. UV–Visible Spectra of Catechol Complexes

**Figure 1:**
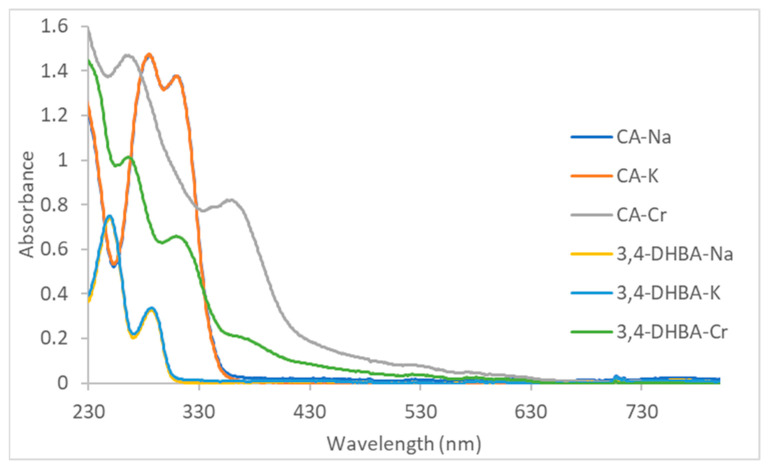
UV–Vis spectra of sodium, potassium, and chromium (tris-catecholate) salts of CA and 3,4-DHBA; at 0.1 mM of ligand. Abbreviations: CA = caffeic acid, 3,4-DHBA = 3,4-dihydroxybenzoic acid.

The UV–vis spectra of the monosodium and monopotassium CA and 3,4-DHBA salts closely resemble those of the free acids reported in the literature (Figure 1) [45,46], which corroborates that under these conditions, only simple salt formation occurs. For CA and 3,4-DHBA sodium and potassium salts, the two strong UV bands are π→π* on the aromatic ring (“Band II”). For alkaline 3,4-dihydroxybenzoic acid, two bands are observed at 250 and 290 nm; in this case, the π system is essentially confined to the aromatic ring with only weak interaction with the carboxyl group, giving a relatively large HOMO–LUMO separation. In alkaline caffeic acid, the corresponding bands appear at 288 and 312 nm. The presence of the vinylene group links the aromatic ring to the carboxyl π system, creating extended conjugation. This increased orbital overlap stabilizes the π* level more strongly than the π level, reduces the HOMO–LUMO gap, and shifts the π→π* transitions to lower energy.

For the caffeic acid–Cr(III) complex, in addition to the intense π–π* transitions below 300 nm, a broad absorption band appears with an onset at approximately 320 nm, a shoulder in the 350–370 nm region, and a long tail extending beyond 400 nm. This feature is assigned to an O(2p)→Cr(III) LMCT transition arising from the catecholate moiety [47]. The 3,4-DHBA–Cr(III) complex displays a similar pattern, with LMCT absorption beginning near 310–330 nm and a weaker shoulder around 335–355 nm, but with considerably less intensity and only a modest extension into the visible region (consistent with the observed brown-green color, more intense for the CA–Cr complex). The difference in band position and intensity between the two systems is consistent with the electronic structures of the ligands: the conjugated vinyl group in caffeic acid increases π-delocalization and enhances donor strength, producing a bathochromic shift and a more intense LMCT band compared to 3,4-DHBA.

Earlier kinetic–mechanistic studies on chromium(III) with 3,4-dihydroxybenzoic acid [48] established that under weakly acidic aqueous conditions (pH < 4), the reaction proceeds in a slow, multistep fashion. Only a 1:1 Cr/ligand complex was detected, formed through initial carboxylate binding, followed by intramolecular catechol chelation. The acidic medium limits ligand deprotonation, and the kinetic inertness of Cr(III) prevents further coordination within the experimental timeframe. Indeed, Cr(III)–catecholate complexes are well known for their kinetic inertness: Sever and Wilker reported that Cr^3+^ titrations with catecholates required up to 30 days for equilibration due to extremely slow ligand exchange [47], consistent with earlier studies on tris(catecholato)–Cr(III) analogs [49] and more recent reports highlighting sluggish substitution in Cr(III)–siderophore complexes [50]. This speciation is therefore relevant to short-term aqueous reactions and biological environments where pH and competing ligands constrain complexation.

By contrast, the present synthesis was performed in mildly alkaline conditions (NaOH present, pH around 8–9) with an excess of ligand (3:1 input ratio), elevated temperature (50 °C), and prolonged equilibration (48 h). Under these circumstances, the carboxylate group is fully deprotonated, and at least one of the catechol hydroxyls is substantially deprotonated, providing strong O,O-donors capable of saturating the Cr(III) coordination sphere. When we later attempted Job’s method at dilute concentrations, the very slow kinetics of Cr(III) led to a gradual spectral evolution; only after approximately three days did the solutions develop the characteristic pattern of the 3:1 complexes. In contrast, when the pre-isolated powders were simply dissolved in water, the spectra characteristic of the 3:1 species appeared immediately, reflecting the stability of the thermodynamic product once formed.

### 2.4. ABTS^+^ Radical Scavenging Activity

**Figure 2:**
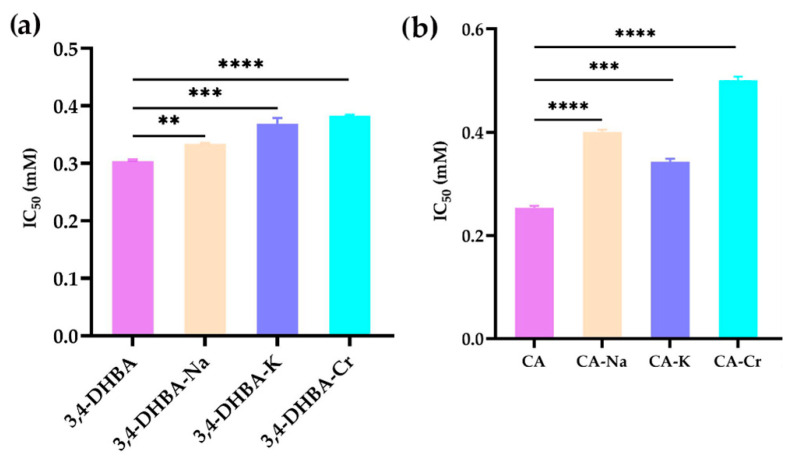
ABTS^+^ radical scavenging activity of Na, K, and Cr 3,4-dihydroxybenzoates (**a**) and caffeates (**b**). The stars denote statistical significance: ** *p* ≤ 0.01, *** *p* ≤ 0.001, **** *p* ≤ 0.0001.

Figure 2 shows the ABTS^+^• radical scavenging activity of 3,4-dihydroxybenzoic acid (3,4-DHBA), caffeic acid (CA), and their complexes with Na, K, and Cr. Compared to 3,4-DHBA, the IC_50_ values for 3,4-DHBA complexed with Na, K, and Cr increased by 10, 21, and 26%, respectively. Similarly, the IC_50_ values for CA and its complexes increased by 58%, 32%, and 98%, respectively. The significant increase in IC_50_ values after metal coordination demonstrates that the ABTS^+^ radical scavenging activity of these compounds decreased upon coordination.

### 2.5. DPPH Radical Scavenging Activity

**Figure 3:**
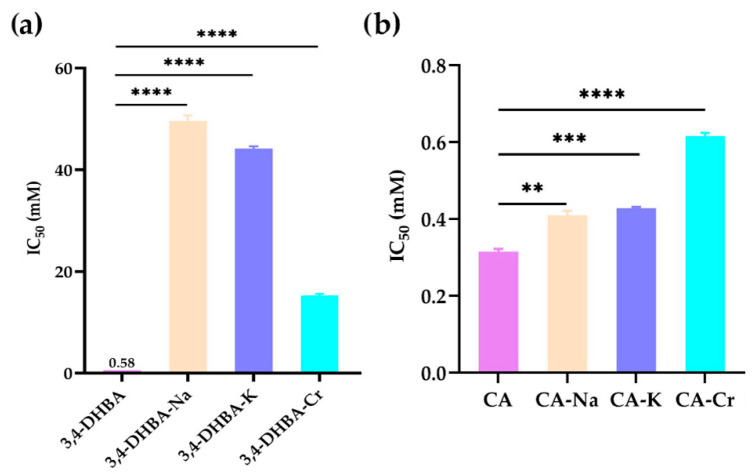
DPPH• radical scavenging activity of Na, K, and Cr, 3,4-dihydroxybenzoates (**a**), and caffeates (**b**). The stars denote statistical significance: ** *p* ≤ 0.01, *** *p* ≤ 0.001, **** *p* ≤ 0.0001.

Figure 3 illustrates the DPPH• radical scavenging activity of 3,4-dihydroxybenzoic acid (3,4-DHBA), caffeic acid (CA), and their complexes with Na, K, and Cr. The IC_50_ values for 3,4-DHBA and its complexes were 0.58, 50, 44, and 15 mM, respectively, while the IC_50_ value for CA and its complexes was 0.32, 0.41, 0.42, and 0.62 mM, respectively. Compared to the free ligands, the IC_50_ value of their salts and complexes was significantly higher, indicating that the DPPH radical scavenging activity of 3,4-DHBA and CA decreased after metal coordination.

### 2.6. Hydroxyl Radical (OH) Scavenging Activity

**Figure 4:**
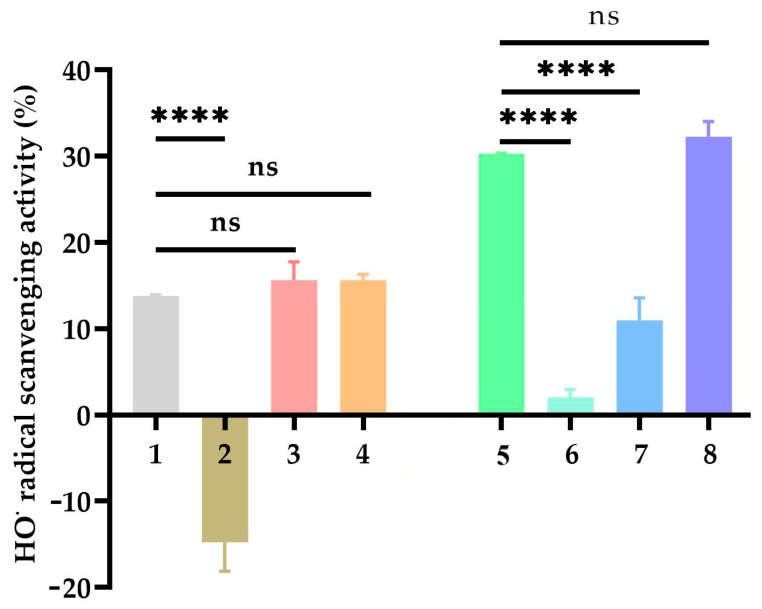
Hydroxyl radical (OH) scavenging activity of Na, K, and Cr 3,4-dihydroxybenzoates and caffeates (1 mM). 1: 3,4-DHBA; 2: 3,4-DHBA-Na; 3: 3,4-DHBA-K; 4: 3,4-DHBA-Cr; 5: CA; 6: CA–Na; 7: CA–K; 8: CA–Cr; The stars denote statistical significance: **** *p* ≤ 0.0001. ns—nonsignificant statistically.

Figure 4 shows the hydroxyl radical scavenging activity (%) of 3,4-DHBA, CA, and their metal complexes. The scavenging activity of 3,4-DHBA alone was moderate, while its Na complex (3,4-DHBA-Na) exhibited negative activity values, indicating that Na coordination actually promoted hydroxyl radical generation. In contrast, the K complex (3,4-DHBA-K) and Cr complex (3,4-DHBA-Cr) showed activities that were not significantly different from 3,4-DHBA, though Cr coordination produced a slight, non-significant increase.

For CA and its complexes, coordination with Na or K significantly decreased the hydroxyl radical scavenging activity compared to CA alone, while Cr coordination preserved the activity at a level similar to that of free CA.

### 2.7. Superoxide Radical (O_2_^−^) Scavenging Activity

**Figure 5:**
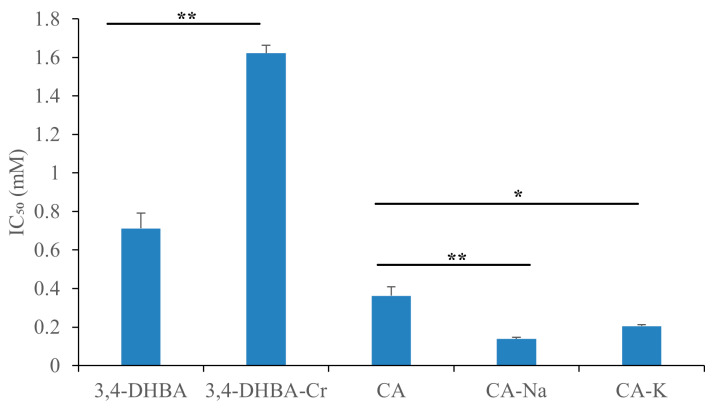
Superoxide radical (O_2_^−^) scavenging activity of Cr 3,4-dihydroxybenzoates and Na and K caffeates. The stars denote statistical significance: * *p* ≤ 0.05, ** *p* ≤ 0.01.

Figure 5 displays the superoxide radical scavenging activity of 3,4-dihydroxybenzoic acid (3,4-DHBA) and caffeic acid (CA) after metal coordination. The IC_50_ values for 3,4-DHBA-Cr were significantly higher than those of 3,4-DHBA, indicating reduced scavenging activity. In contrast, the coordination of Na and K enhanced the superoxide radical scavenging activity of CA. Nevertheless, at the applied concentration range (≈0.5 mM), 3,4-DHBA-Na, 3,4-DHBA-K, and CA–Cr displayed virtually no detectable superoxide radical scavenging activity. When tested at higher concentrations, these compounds yielded negative values of superoxide radical scavenging activity, indicating a shift toward pro-oxidative behavior rather than antioxidant activity. Accordingly, these complexes were grouped in the tables (Table 4 and Table 5) together with the corresponding free acids to highlight this pro-oxidative effect. The alkaline salts of 3,4-DHBA produced only small negative SRSA values (approximately −2% to −7% across 3–8 mM). These modest decreases are most reasonably interpreted as assay artifacts, likely due to oxidation or destabilization of the reduced formazan product, rather than reflecting true pro-oxidative activity. In contrast, the CA–Cr complex exhibited consistently large negative SRSA values, reaching nearly −27% at a concentration of 0.4 mM. The magnitude of this effect strongly suggests a redox process associated with the chromium center, which may facilitate the generation of reactive oxygen species under the tested conditions.

A striking aspect of our findings is that the structural features that make caffeic acid a highly efficient superoxide scavenger in its free form also render it particularly susceptible to a shift toward pro-oxidant behavior upon coordination with chromium. Caffeic acid possesses an extended conjugated π-system arising from the cinnamic side chain, which allows the unpaired electron of the phenoxyl radical to delocalize over both the catechol ring and the vinyl–carboxyl moiety. This electronic stabilization lowers the bond dissociation energy of the phenolic O–H groups, enhancing their hydrogen- and electron-donating capacity, which accounts for the strong antioxidant activity of the free acid. However, when coordinated to chromium, this same conjugated system provides an efficient pathway for electron transfer between the ligand and the metal center. In this context, the redox-active chromium can undergo cycling reactions that generate reactive oxygen species, shifting the overall effect from antioxidant to pro-oxidant. This mechanistic distinction explains why free caffeic acid is among the most potent scavengers in the series, while its chromium complex exhibits the most pronounced pro-oxidative behavior.

### 2.8. Ferric-Reducing Antioxidant Power

**Figure 6:**
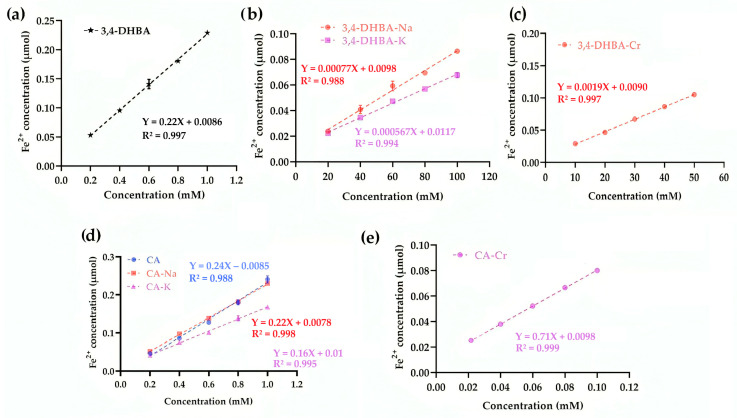
Ferric-reducing antioxidant power of 3,4-DHBA (**a**) and its Na, K, and Cr derivatives (**b**,**c**); caffeic acid and its Na, K, and Cr derivatives (**d**,**e**).

Unlike in the superoxide scavenging assay, where CA–Cr displayed pro-oxidant behavior, in the FRAP assay, the same complex manifested as a markedly stronger reductant. Figure 6 presents the ferric-reducing antioxidant power (FRAP) of 3,4-DHBA, CA, and their complexes. For 3,4-DHBA, coordination or salt formation sharply reduced activity (slopes: 0.22 for the free acid vs. ≤0.0019 for its derivatives). In contrast, CA–Cr exhibited a striking ~196% increase in slope compared to CA (0.71 vs. 0.24), while CA–Na and CA–K showed only minor decreases. This contrasting behavior reflects structural differences: the conjugated double bond in CA promotes electron transfer and amplifies the effect of chromium coordination. Thus, whereas Cr diminished the reducing power of 3,4-DHBA, it markedly enhanced that of CA.

This apparent discrepancy between FRAP and SRSA assays arises from their different principles: FRAP reflects the capacity of a compound to act as an electron donor to a stable acceptor (Fe^3+^), whereas the SRSA assay monitors the balance between superoxide generation and removal in the presence of reactive intermediates. Our hypothesis is that in the CA–Cr complex, the conjugated π-system of caffeic acid facilitates efficient electron transfer to the chromium center. This process enhances the reducing power observed in the FRAP assay, while simultaneously enabling chromium redox cycling, which promotes reactive oxygen species formation and accounts for the negative SRSA values.

### 2.9. Cupric Reducing Antioxidant Activity

**Figure 7:**
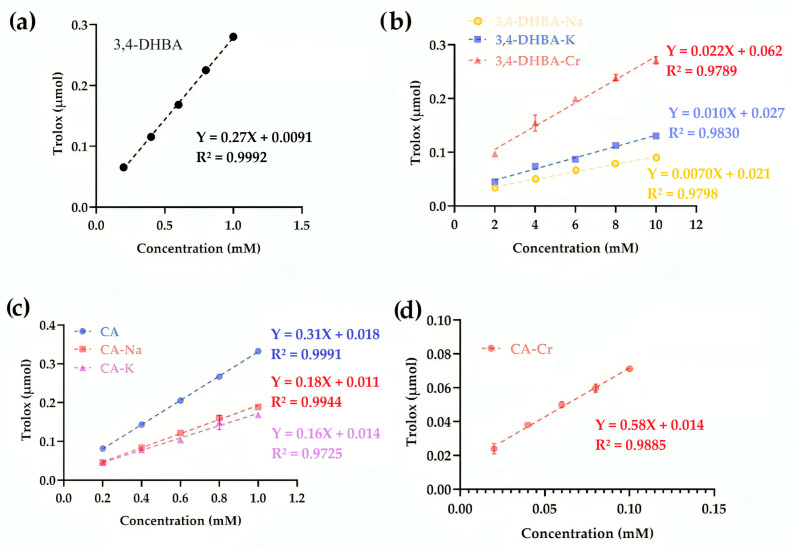
CUPRAC activity of 3,4-dihydroxybenzoic acid (**a**) and its derivatives: Na, K salts, and Cr complex (**b**). CUPRAC activity of caffeic acid, CA–Na, and CA–K (**c**). CUPRAC activity of CA–Cr (**d**).

Figure 7 illustrates the cupric reducing antioxidant capacity (CUPRAC) of 3,4-DHBA, CA, and their complexes. For 3,4-DHBA, the slope values were 0.27 (free acid), 0.007 (Na), 0.01 (K), and 0.022 (Cr), confirming that coordination or salt formation markedly lowered its reducing activity. By contrast, CA exhibited a slope of 0.31, while CA–Na and CA–K decreased to 0.18 and 0.16, respectively. Strikingly, CA–Cr displayed a slope of 0.66, more than double that of free CA, indicating that chromium coordination substantially enhanced electron transfer to Cu^2+^. This divergence parallels the FRAP results, in which CA–Cr also showed greatly elevated reducing power, and contrasts with the SRSA assay, where CA–Cr appeared pro-oxidant. The conjugated catechol–propenoic acid structure of CA provides an efficient electron-donating framework, which upon Cr coordination amplifies reducing activity in single-electron transfer assays (FRAP, CUPRAC). However, the same electron transfer enables redox cycling under aerobic conditions, explaining the negative SRSA values.

### 2.10. Inhibition of Linoleic Acid Peroxidation

**Figure 8:**
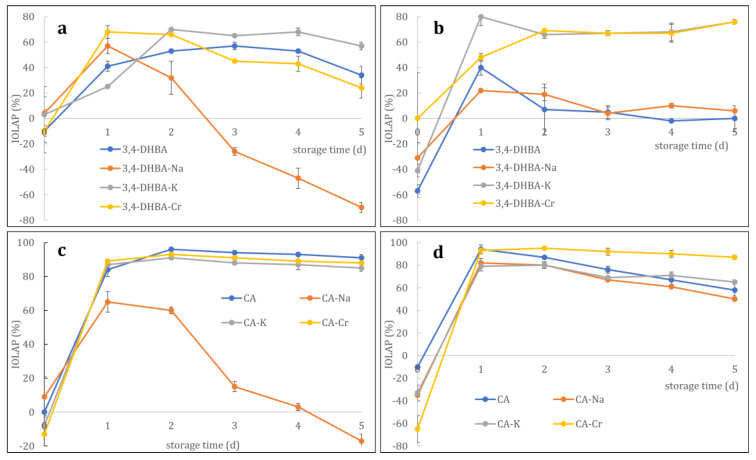
Linoleic acid peroxidation of Na, K, and Cr 3,4-dihydroxybenzoates and caffeates. 5 μM: (**a**) and (**c**). 10 μM: (**b**) and (**d**).

Figure 8 illustrates the inhibition of linoleic acid peroxidation (IOLAP) of 3,4-DHBA, CA, their Na and K salts, and the Cr complex with increasing incubation periods at 40 °C. The inhibitory effects of 3,4-dihydroxybenzoic acid (3,4-DHBA) and caffeic acid (CA), as well as their sodium, potassium, and chromium salts/complexes, on linoleic acid peroxidation were assessed at 5 and 10 μM concentrations.

At 5 μM (Figure 8a), 3,4-DHBA alone exhibited a moderate increase in inhibition over time, reaching approximately 50% by day 5. The sodium salt initially increased but then sharply declined, becoming a pro-oxidant. In contrast, the potassium salt displayed the strongest and most sustained inhibition, peaking at ~70% on day 2 and remaining above 50% thereafter. The chromium complex also showed stable inhibitory activity, maintaining ~30% by day 5.

At 10 μM (Figure 8b), free 3,4-DHBA initially showed pro-oxidant behavior (−50%) but rapidly increased to ~30% inhibition on day 1, followed by a gradual decline to baseline. The sodium salt transitioned from weak inhibition to near inactivity, while the potassium salt maintained strong activity (~70–80%). The chromium complex exhibited delayed but consistent inhibition, reaching a plateau of approximately 70% by day 2.

For CA and its derivatives at 5 μM (Figure 8c), all compounds except the sodium salt showed rapid and sustained inhibition above 80% after day 1, with minimal decline. The sodium salt exhibited strong inhibition on day 1 (60%) but progressively lost activity, ultimately becoming a pro-oxidant (−20%) by day 5.

At 10 μM (Figure 8d), CA itself and its derivatives (K and Cr salts) all exhibited near-complete inhibition (~100%) from day 1 onward, with only minor declines (10–20%) over time. In contrast, the sodium salt displayed the lowest stability, falling to ~50% inhibition by day 5.

Together, these results indicate that potassium and chromium salts generally stabilize the antioxidant capacity of both phenolic acids, while sodium salts destabilize their activity, often promoting pro-oxidant effects during prolonged incubation. Chromium complexes of both 3,4-dihydroxybenzoic and caffeic acids generally exhibited more sustained inhibition of linoleic acid peroxidation, comparable to or better than the free ligands.

## 3. Discussion

Antioxidant properties are crucial in biochemical and pharmaceutical contexts because they neutralize free radicals, which can cause cellular damage through oxidative stress [51]. Oxidative stress has been associated with the pathogenesis of numerous chronic diseases, including cardiovascular disorders, cancer, diabetes, and neurodegenerative conditions [52]. Antioxidants help protect biomolecules, such as lipids, proteins, and DNA, from oxidative damage, thereby maintaining cellular integrity and preventing disease progression [53]. In pharmaceutical applications, compounds with strong antioxidant properties are gaining attention due to their potential to develop therapies for alleviating oxidative stress-related conditions, as well as formulations to prolong product shelf life by preventing oxidation [54]. This work investigated the antioxidant properties of 3,4-DHBA, CA, and their complexes, including ABTS^+^, DPPH, OH, O_2_^−^, FRAP, CUPRAC, and IOLAP. These findings of ABTS^+^ and DPPH• assays showed that Na, K, and Cr coordination reduced the ABTS^+^• and DPPH• radical scavenging activity of both 3,4-DHBA and CA. Kalinowska et al. (2022) demonstrated that Cu and Fe coordination reduced the ABTS^+^ and DPPH radical scavenging activity of chlorogenic acid [38]. Nevertheless, the OH radical scavenging activity of 3,4-DHBA after K and Cr coordination was higher than that of 3,4-DHBA. The OH radical scavenging activity of CA after Cr coordination was also higher than that of CA. Moreover, the O_2_^−^ radical scavenging activity of CA after Na and K coordination was higher than that of CA, whereas the O_2_^−^ radical scavenging activity of CA after Cr coordination was lower. On the contrary, it was found that the FRAP and CUPRAC values of CA after Cr coordination were higher than those of CA.

The different results between ABTS^+^, DPPH, OH, O_2_^−^, FRAP, and CUPRAC may be attributed to the type of reaction mechanisms. ABTS^+^, DPPH, O_2_^−^ and OH assays, which utilize both hydrogen atom transfer (HAT) and single-electron transfer (SET), are widely used to measure the antioxidant capacity of substances [55]. The FRAP and CUPRAC assays operate on a single-electron transfer mechanism [56,57]. These assays assess the antioxidant’s ability to donate electrons or hydrogen atoms to neutralize harmful free radicals. Each assay offers unique insights and is influenced by specific environmental factors such as solvent type and pH levels. For example, HAT reactions are generally robust across different solvents and pH values, while SET reactions are significantly impacted by the environment’s acidity [58]. Furthermore, although ABTS^+^, DPPH, O_2_^−^ and OH assays share both HAT and SET mechanisms, their results differed, likely due to variations in solubility and reaction kinetics in the respective media. The ABTS^+^• and DPPH• assays are conducted in methanol, whereas the O_2_•^−^ and •OH assays are performed in phosphate-buffered saline (PBS), and these solvent differences influence electron transfer efficiency. In addition, the coordination of metal ions can modify redox behavior differently in each system: in organic media, complexation may reduce the availability of free phenolic sites and lower scavenging efficiency, whereas in aqueous radical-generating systems, the redox-active metal center can participate in electron-transfer processes, either enhancing or suppressing overall antioxidant activity. Pro-oxidant activity refers to the ability of certain substances to promote the generation of free radicals or reactive oxygen species, which can cause oxidative damage to cells and tissues [59]. Under specific conditions, compounds that generally act as antioxidants can exhibit pro-oxidant behavior, especially when they interact with metal ions or when present in high concentrations [60]. This pro-oxidant activity can contribute to cellular damage, mutagenesis, and the progression of various diseases [61].

Linoleic acid peroxidation is a process in which the polyunsaturated linoleic acid undergoes oxidative degradation, typically initiated by free radicals or reactive oxygen species [62]. This leads to unstable lipid peroxide formation, which can decompose into secondary toxic products that further damage cellular membranes, proteins, and DNA [63,64]. Linoleic acid peroxidation is often used as an experimental model to assess the compound’s pro-oxidant or antioxidant activity, as it helps understand the dynamics of lipid oxidation in biological systems. These results showed that the inhibition of linoleic acid peroxidation in 3,4-DHBA and CA after K and Cr coordination at 10 μM was maintained or slightly increased compared to free ligands. This indicates that K and Cr complexes of 3,4-DHBA and CA may exhibit improved oxidative stability, which could make them useful as lipid-protective agents in biological or food systems.

**Figure 9:**
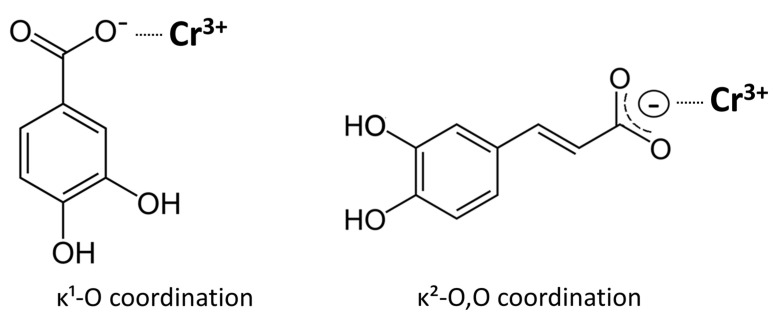
Proposed coordination modes of Cr(III) with caffeic acid (CA) and 3,4-dihydroxybenzoic acid (3,4-DHBA), based on FTIR spectral features and ligand-to-metal ratios. The schemes depict only the plausible ligand–metal interactions rather than the complete coordination environment, which cannot be unambiguously assigned without structural data. In the caffeate complex, the carboxylate group coordinates in a bidentate (κ^2^-O,O′) manner, whereas in the 3,4-dihydroxybenzoate complex, the carboxylate group binds predominantly in a monodentate (κ^1^-O) fashion. The broadening of the catechol ν(OH) bands is attributed to hydrogen bonding involving coordinated water molecules rather than direct metal coordination.

In this study, several possible coordination modes were considered for the chromium complexes. Examination of the FTIR spectra clearly indicates that the carboxylate groups are actively involved in coordination, as shown by the characteristic shifts and splitting of the ν(C=O) and ν(C–O) bands. Elemental analysis excludes models requiring deprotonation of the phenolic hydroxyl groups. Elemental and ash analyses confirm the absence of sodium counter-ions, consistent with deprotonation limited to the carboxyl group of each ligand and the formation of neutral tris(carboxylato)chromium(III) complexes. The FTIR spectra show intact O–H stretching bands for both complexes, confirming that the catechol hydroxyl groups remain protonated and do not participate in coordination; the observed broadening is attributable to hydrogen bonding, most likely involving water molecules present in the complexes. This interpretation agrees with the synthetic conditions, since the complexes were prepared from monosodium salts of the ligands, providing a mildly basic medium that favors carboxylate deprotonation but not phenolic ionization. Consequently, the overall composition corresponds to neutral chromium(III) complexes containing three monoanionic ligands. Thus, the spectroscopic and analytical data consistently show that coordination in both chromium complexes occurs predominantly through the carboxylate groups (Figure 9). For caffeic acid, the FTIR features are consistent with bidentate κ^2^-O,O′ coordination of the carboxylate group. In the Cr(III)–3,4-dihydroxybenzoate complex, the spectra indicate primarily monodentate κ^1^-O carboxylate binding. The visible broadening and slight shifts of the ν(OH) band near 3500 cm^−1^ and the ν(C–O) band around 1250 cm^−1^ are best explained by hydrogen bonding, likely involving water molecules associated with the complexes.

The observed differences in redox behavior between the two Cr(III) complexes can be rationalized by differences in carboxylate coordination mode and the electronic characteristics of the ligand framework. The FTIR spectra show a smaller Δν(COO^−^) value for the Cr(III)–caffeate complex, consistent with a more chelate-like, bidentate coordination of the carboxylate group, whereas the larger Δν observed for the Cr(III)–3,4-dihydroxybenzoate complex indicates a monodentate or predominantly ionic interaction. These variations in carboxylate binding influence the extent of metal–ligand orbital overlap and thus the efficiency of ligand-to-metal charge transfer (LMCT).

The conjugated –CH=CH–COO^−^ side chain of caffeic acid extends the π-system and promotes delocalization of electron density toward the Cr(III) center. Upon coordination, this framework enables low-energy O(2p) → Cr(3d) LMCT transitions, manifested as a more intense and bathochromically shifted UV–vis absorption feature for the Cr–CA complex. Enhanced electronic communication between ligand and metal favors efficient electron transfer, explaining both the higher reducing power observed in single-electron transfer (SET) assays (FRAP, CUPRAC) and the partial pro-oxidant response under superoxide-generating conditions (SRSA).

In contrast, the less conjugated benzoate framework of 3,4-dihydroxybenzoic acid, combined with its monodentate carboxylate coordination, affords weaker orbital coupling and less efficient LMCT. As a result, this complex exhibits lower overall redox activity, reflected in its reduced performance in SET-based assays and the absence of measurable pro-oxidant behavior.

## 4. Materials and Methods

### 4.1. Materials

ABTS (2,2-azino-bis(3-ethylbenzothiazoline-6-sulfonic acid)) and β-nicotinamide adenine dinucleotide (β-NADH) were purchased from Tokyo Chemical Industry Co., LTD. Potassium persulfate (p.p.a.) was bought from Fluka. Caffeic acid (CA), DPPH (2,2-diphenyl-1-picrylhydrazyl), 2-deoxy-D-ribose (DDR), ethylenediaminetetraacetic acid (EDTA), hydrogen peroxide (H_2_O_2_), iron(III) chloride (FeCl_3_), iron(II) sulfate (FeSO_4_), thiobarbituric acid (TBA), trichloroacetic acid (TCA), nitroblue tetrazolium (NBT), phenazine methosulfate (PM), 2,4,5-tris(2-pyridyl)-s-triazine) (TPTZ), ammonium acetate, neocuproine, Trolox, linoleic acid, and Tween-20 were bought from Sigma-Aldrich. NH_4_SCN, CuCl_2_, ascorbic acid, and CH_3_COONa were purchased from Chempur (Piekary Slaskie, Poland). All the compounds were of analytical grade purity (or provided with a purity statement at each). All chemicals were used as received without further purification. 3,4-DHBA and caffeic acids (98%), alkali metal hydroxides, and chromium(III) chloride were purchased from Sigma Aldrich (St. Louis, Missouri, USA).

### 4.2. Synthesis of Complexes

Synthesis of sodium and potassium salts

Portions of 3,4-DHBA and caffeic acids (about 0.2 g) were dissolved in a solution of alkali metal hydroxide (NaOH, KOH) at a concentration of 0.1 mol/dm^3^ in a stoichiometric amount (1:1). The solution was heated to 80 °C and stirred until the acids dissolved. After the reaction, the solution was slowly evaporated at room temperature. During the evaporation of water, crystalline substances were obtained, which were placed in an oven for 48 h at 102 °C to remove residual moisture.

Synthesis of chromium(III) complexes

A portion of 3,4-DHBA and caffeic acids (about 0.2 g) was dissolved in aqueous sodium hydroxide solution in a stoichiometric amount (1:1). An aqueous solution of chromium(III) chloride was added to the solution at a concentration of 0.1 mol/dm^3^ in a stoichiometric amount of 3:1 (ligand/metal). The whole mixture was stirred in a shaker for 2 h at 50 °C, leading to the green precipitates. The turbid solutions were left for 48 h to precipitate. The resulting complexes were drained using filters and then washed to elute residual chlorides. The precipitates were dried at room temperature in a desiccator for 72 h and then stored in airtight vessels.

The synthesis was repeated three times, and each batch of the obtained compounds was subjected to preliminary FTIR analysis.

### 4.3. The FTIR Spectra

FTIR spectra were recorded using KBr pellets in the range of 400–4000 cm^−1^ on an Alfa spectrometer (Bruker, Billerica, MA, USA).

### 4.4. Ash Content

Approximately 50 mg of the dried complex was placed in pre-weighed porcelain crucibles, dried to a constant mass at 110 °C, and then gradually heated to 650–700 °C in air and ashed to a constant weight, with cooling in a desiccator between heating cycles.

### 4.5. Elemental Analysis

Elemental composition (C, H, N, S) of the samples was determined using an Elementar Vario Micro Cube analyzer operating in dynamic combustion mode. Approximately 2 mg of each sample was used in each replicate. Each sample was analyzed in triplicate, and the obtained peak areas were integrated automatically. The system operated with helium as the carrier gas and high-temperature combustion followed by gas chromatographic separation and thermal conductivity detection. Sulfanilamide was used as the calibration standard.

### 4.6. UV–Vis Spectroscopy

UV–vis absorption spectra were recorded in standard macro quartz cuvettes (QS, 1 cm path length; Hellma Analytics, Müllheim, Germany), in water, using a Vernier SpectroVis Plus spectrophotometer/fluorimeter (Logger Pro 3 software version 3.8.2, Vernier Software & Technology, Beaverton, OR, USA). Spectra were collected over the 200–800 nm range.

### 4.7. ABTS^+^ Radical Scavenging Activity

ABTS powder (0.0384 g) and 0.0066 g of potassium persulfate were added to 10 mL of distilled water, and the mixture was placed in a dark environment at room temperature for 16 h. Then, the mixture was diluted with methanol to achieve an absorbance of 0.75 at 734 nm. 100 μL of 3,4-dihydroxybenzoic acid, caffeic acid, and their complex was mixed with 3 mL of the mixture and incubated at room temperature for 8 min. The absorbance was determined at 734 nm using the UV–VIS spectrophotometer. The ABTS^+^ radical scavenging capacity was expressed as a concentration of 50% ABTS^+^• radical scavenging (IC_50_) [14,35,65].

### 4.8. DPPH Radical Scavenging Activity

DPPH (0.0198 g) was dissolved in 80% methanol, and the resulting DPPH solution was placed in a dark environment at room temperature for 3 h. Then, the DPPH solution was diluted with 80% methanol to achieve an absorbance of 0.75 at 516 nm. 100 μL aliquots of 3,4-dihydroxybenzoic acid, caffeic acid, and their complexes were mixed with 3 mL DPPH solution. The absorbance of the mixture after 30 min. of incubation was determined at 516 nm. The DPPH radical scavenging capacity was quantified as the IC_50_ value, representing the concentration of the tested compound required to achieve 50% scavenging of DPPH radicals [14,35,66]. Absorbance of a blank containing the compound in methanol was subtracted.

### 4.9. Hydroxyl Radical (OH) Scavenging Activity

Hydroxyl radical solution (HRS) was prepared by mixing 28 mM DDR in PBS (pH = 7.4), 1 mM EDTA, 10 mM H_2_O_2_, 1 mM FeCl_3,_ and 1 mM ascorbic acid in a volumetric ratio of 1:1:1:1:1. 500 μL of 3,4-dihydroxybenzoic acid, caffeic acid, and their complex was added to the HRS, and the mixture was incubated at 37 °C for 60 min. After incubation, 1 mL of 1% TBA and 1 mL of 2.8% TCA were added to the mixture and heated at 100 °C for 20 min. The absorbance of the final mixture was determined at 532 nm [65,67]. Absorbance of a blank containing the compound in buffer was subtracted.

### 4.10. Superoxide Radical (O_2_^−^) Scavenging Activity

Superoxide radical solution (SRS) was prepared by mixing 0.5 mL of 1.35 mM NADH, 0.5 mL of 0.15 mM NT, and 0.5 mL of 0.12 mM PM. 500 μL of 3,4-dihydroxybenzoic acid, caffeic acid, and their complex was added to the SRS, and the absorbance of the mixture after 5 min of incubation was determined at 650 nm. The superoxide radical scavenging capacity was expressed as the concentration required for 50% superoxide radical scavenging (IC_50_).

### 4.11. Ferric-Reducing Antioxidant Power (FRAP)

3,4-dihydroxybenzoic acid, caffeic acid, and their complex (100 μL) were mixed with the FRAP reagent, which contained 2.5 mL of pH = 3.6 acetate buffer, 250 μL of 10 mM TPTZ, and 250 μL of 20 mM FeCl_3_. The mixture was incubated at 37 °C for 15 min, and their absorbance after incubation was determined at 593 nm. The FRAP capacity was expressed as Fe^2+^ equivalents [35,58,68]. Absorbance of a blank containing the compound in buffer was subtracted.

### 4.12. Cupric Reducing Antioxidant Activity (CUPRAC)

3,4-dihydroxybenzoic acid, caffeic acid, and their complex (100 μL) were mixed with the CUPRAC reagent, which contained 0.8 mL of 10 mM CuCl_2_, 800 μL of 7.5 mM neocuproine in methanol, and 800 μL of 1 M ammonium acetate buffer (pH = 7). The mixture was incubated at room temperature in a dark environment for 60 min, and its absorbance after incubation was determined at 450 nm. The CUPRAC was expressed as Trolox equivalents, based on a calibration curve prepared from standard Trolox solutions [58,68]. Absorbance of a blank containing the compound in buffer and methanol was subtracted.

### 4.13. Measurement of Inhibition of Linoleic Acid Peroxidation

3,4-Dihydroxybenzoic acid, caffeic acid, and their complexes (1 mL) were mixed with 1.5 mL of linoleic acid containing 312 μL of LA, 256 μL of Tween-20, and 200 μL of 50 mM PBS (pH 7.0). The mixture was incubated at 40 °C for 5 days, and 100 μL aliquots were withdrawn daily. Each aliquot (100 μL) was combined with 50 μL of 30% NH_4_SCN and 4.7 mL of 80% methanol, then incubated at room temperature for 3 min. After incubation, 50 μL of 20 mM FeCl_2_ was added, and the absorbance of the final mixture was immediately measured at 500 nm [67,69]. Absorbance of a blank containing the compound in buffer was subtracted.

### 4.14. Statistical Analysis

The obtained data on the antioxidant activities of Na(I), K(I), Cr(III), and Fe(III) 3,4-dihydroxybenzoates and vaffeates were presented as mean ± standard deviation values. All figures were prepared using GraphPad Prism 9. Significant differences were computed using the one-way analysis of variance (ANOVA) with Tukey’s post hoc test in IBM SPSS Statistics 26 software.

## 5. Conclusions

This study demonstrates that antioxidant activity extends beyond hydroxyl groups (–OH) and is significantly influenced by metal complexation. Coordination with metal ions of high ionic potential and favorable charge-to-radius ratios enhances electron delocalization, stabilizing free radicals and improving antioxidant efficiency, particularly in aromatic systems. Complexation increases charge redistribution, further reinforcing redox stability. The observed correlations align with existing literature, confirming that metal coordination modulates antioxidant performance [30,69,70,71]. These findings suggest potential applications in food preservation and functional food formulations, where metal–antioxidant complexes may offer superior stability and efficacy. Moreover, to determine whether a given compound stabilizes free radicals, scavenges free radicals, or possesses antioxidant activity, it is essential to analyze its structure, spectroscopic data, and HOMO–LUMO gaps and infer the stabilization of the electronic system within the aromatic ring or aromatic system. However, assessing biological activity requires considering additional factors, such as lipophilicity, interactions with enzymes—including membrane-bound enzymes and cytochromes—regulation of cellular signaling pathways, regulation capacity, as well as permeability through biological barriers, metabolism, and, in effect, the concentrations and forms that reach the cells.

The results revealed that metal coordination significantly modulates the antioxidant and pro-oxidant activities of both polyphenolic compounds. While Na, K, and Cr reduced the ABTS^+^ and DPPH radical scavenging activities of both ligands, Cr coordination maintained or slightly enhanced hydroxyl radical scavenging compared to the free acids. Moreover, Cr markedly increased the reducing capacity (FRAP and CUPRAC) of CA but decreased that of 3,4-DHBA. The pro-oxidant behavior of 3,4-DHBA, CA, and their complexes was most evident in the superoxide scavenging and linoleic acid peroxidation assays. For 3,4-DHBA, Na and K salts produced only small negative SRSA values, which are best interpreted as assay artifacts. In contrast, Cr coordination largely maintained or slightly reduced activity, indicating limited engagement in redox cycling. In contrast, the CA–Cr complex consistently showed strongly negative SRSA values, demonstrating pronounced pro-oxidant activity. This divergence is rooted in structural differences: the catechol group in 3,4-DHBA stabilizes coordinated metals but does not provide an efficient electron-transfer pathway, thereby restraining redox cycling. In contrast, caffeic acid, with its extended conjugated π-system, enables the delocalization of the phenoxyl radical across both the aromatic ring and cinnamic side chain. This electronic structure makes CA a highly effective antioxidant in its free form; however, when coordinated to a redox-active metal such as chromium, it also provides an efficient channel for electron transfer. In the FRAP and CUPRAC assays, this manifests as greatly enhanced reducing power, while in the superoxide assay, it drives chromium redox cycling and ROS generation, explaining the shift from antioxidant to pro-oxidant behavior.

A deeper analysis of these findings highlights several mechanistic insights: (1) Complexation of caffeic acid with Cr(III) enhances its antioxidant capacity by delocalizing electronic charge, stabilizing the hydroxyl groups, and lowering the overall energy of the system. Importantly, in the Cr–CA complex, free hydroxyl groups remain available because coordination occurs primarily through the carboxyl group. (2) By contrast, 3,4-DHBA–Cr(III) complexes show reduced antioxidant properties because both the carboxylate and hydroxyl donors participate in coordination, thereby blocking groups critical for direct radical scavenging. IR spectra and steric considerations support these proposed binding modes, although definitive confirmation would require X-ray crystallography, ideally complemented by solution studies that consider intra- and intermolecular interactions. (3) Formation of Na and K salts with polyphenols perturbs the electronic charge distribution of the ligands, diminishing their antioxidant potential. (4) Caffeic acid and its Cr(III) complexes consistently outperform 3,4-DHBA and its derivatives in antioxidant capacity. This difference can be attributed to the extended conjugated π-system in CA, which further delocalizes electronic charge, stabilizes the antioxidant, and lowers its energy—a conclusion in line with earlier reports.

Our previous studies also indicated that the effectiveness of antioxidants depends not only on the number and position of hydroxyl and methoxy groups but also on additional structural factors. Enhanced reducing power is associated with: (1) greater charge delocalization and reduced energy of the ligand, (2) extension of the conjugated double-bond system, (3) a wider energy gap between the antioxidant and the radical, and (4) the presence of additional delocalized aromatic rings. Moreover, we earlier observed that complexation with metals of high ionic potential, such as Fe(III), Al(III), and Ln(III), markedly increases the reducing activity of phenolic ligands. The present findings for CA–Cr(III) complexes corroborate these trends.

Together, these results provide a mechanistic framework for optimizing metal–polyphenol complexes in pharmaceutical and nutraceutical applications. Properly designed complexes may not only mitigate oxidative stress but also extend product stability. Future research should move beyond chemical assays to cellular and in vivo models, where the interplay of lipophilicity, redox potential, enzymatic interactions, signaling regulation, permeability, and metabolism ultimately determines the net antioxidant or pro-oxidant outcomes.

## Figures and Tables

**Table 3:** Comparison of Theoretical and Experimental Composition of Chromium Complexes.

Formula	MW	%C	%H	%Ash	C/H
CA–Cr Cr(C_9_H_7_O_4_)_3_·9H_2_O	751.58	Theory: 43.14	Theory: 5.23	Theory: 10.11	Theory: 8.25
		Found: 43.04	Found: 5.16	Found: 12.97	Found: 8.35
3,4-DHBA-Cr [Cr(C_7_H_5_O_4_)_3_]·3H_2_O	565.36	Theory: 44.61	Theory: 3.74	Theory: 13.44	Theory: 11.92
		Found: 44.06	Found: 3.86	Found: 15.30	Found: 11.42

**Table 4:** Superoxide radical (O_2_•^−^) scavenging/pro-oxidant activity of Na and K 3,4-dihydroxybenzoates. SRSA, superoxide radical scavenging activity (%); Conc., concentration (mM). Negative values denote pro-oxidant activity (bolded).

3,4-DHBA		3,4-DHBA-Na		3,4-DHBA-K	
Conc. (mM)	SRSA (%)	Conc. (mM)	SRSA (%)	Conc. (mM)	SRSA (%)
0.3	12 ± 4	3	−2.5 ± 0.2	3	**−6.9 ± 0.9**
0.4	20 ± 6	4	−2.7 ± 1.9	4	**−7.0 ± 2.3**
0.5	29 ± 2	5	−1.9 ± 0.4	5	**−5.0 ± 3.7**
0.6	40 ± 6	6	−3.0 ± 1.9	6	**−6.2 ± 0.9**
0.7	48 ± 1	7	−5.7 ± 3.1	7	**−7.2 ± 1.5**
0.8	55 ± 5	8	−6.2 ± 2.6	8	**−6.4 ± 1.6**

**Table 5:** Superoxide radical (O_2_•^−^) scavenging/pro-oxidant activity of Cr–caffeate. SRSA, superoxide radical scavenging activity (%); Conc., concentration (mM). Negative values denote pro-oxidant activity (bolded).

CA		CA–Cr	
Conc. (mM)	SRSA (%)	Conc. (mM)	SRSA (%)
0.1	14.9 ± 0.2	0.2	**−18 ± 4**
0.12	28.0 ± 1.0	0.3	**−21 ± 2**
0.14	46.9 ± 9.4	0.4	**−27 ± 1**
0.16	59.4 ± 10.6	0.5	**−25 ± 1**
0.18	67.1 ± 4.7	0.6	**−23 ± 5**
0.20	73.8 ± 6.0	0.7	**−23 ± 1**

## Data Availability

The metadata will be available from the authors on request.

## Supplementary Materials

The following supporting information can be downloaded at https://www.mdpi.com/article/10.3390/molecules30224467/s1, Figure S1. FT-IR spectra of CA (a), CA-Na (b), CA-K, CA-Cr(III) in the range of 4000–400 cm^−1^; Figure S2. FT-IR spectra of 3,4-DHBA (a), 3,4-DHBA-Na (b), 3,4-DHBA-K, 3,4-DHBA-Cr(III) in the range of 4000–400 cm^−1^.

## Abbreviations

The following abbreviations are used in this manuscript:

3,4-DHBA	3,4-Dihydroxybenzoic Acid
CA	Caffeic Acid
ABTS	2,2′-Azino-bis(3-ethylbenzothiazoline-6-sulfonic acid)
DPPH	2,2-Diphenyl-1-picrylhydrazyl
OH	Hydroxyl radical
O_2_^−^	Superoxide radical
FRAP	Ferric-Reducing Antioxidant Power
CUPRAC	Cupric Reducing Antioxidant Capacity
IOLAP	Inhibition of Linoleic Acid Peroxidation
EDTA	Ethylenediaminetetraacetic Acid
H_2_O_2_	Hydrogen Peroxide
FeCl_3_	Iron(III) Chloride
FeSO_4_	Iron(II) Sulfate
TBA	Thiobarbituric Acid
TCA	Trichloroacetic Acid
NT	Nitroblue Tetrazolium
PM	Phenazine Methosulfate
TPTZ	2,4,5-Tris(2-pyridyl)-s-triazine
DDR	2-Deoxy-D-ribose
NADH	β-Nicotinamide Adenine Dinucleotide (reduced form)
Na	Sodium
K	Potassium
Cr	Chromium
MeOH	Methanol
PBS	Phosphate-Buffered Saline
AMR	Not defined in-text; likely Ammonium Reagent or similar
EC_50_	Effective Concentration for 50% inhibition or activity
IC_50_	Inhibitory Concentration for 50% activity
