# Supplementary material for: Effective Antioxidants as Plausible Ligands in Chromium(III) Supplementation: How Complexation Modulates Catechol-Based Polyphenols
Source: Molecules. 2025 Nov 19;30(22):4467. doi: 10.3390/molecules30224467 (PMC12655245; doi:10.3390/molecules30224467)
Supplement: Supplementary file 1 [file molecules-30-04467-s001.zip › molecules-3873951-supplementary.pdf]

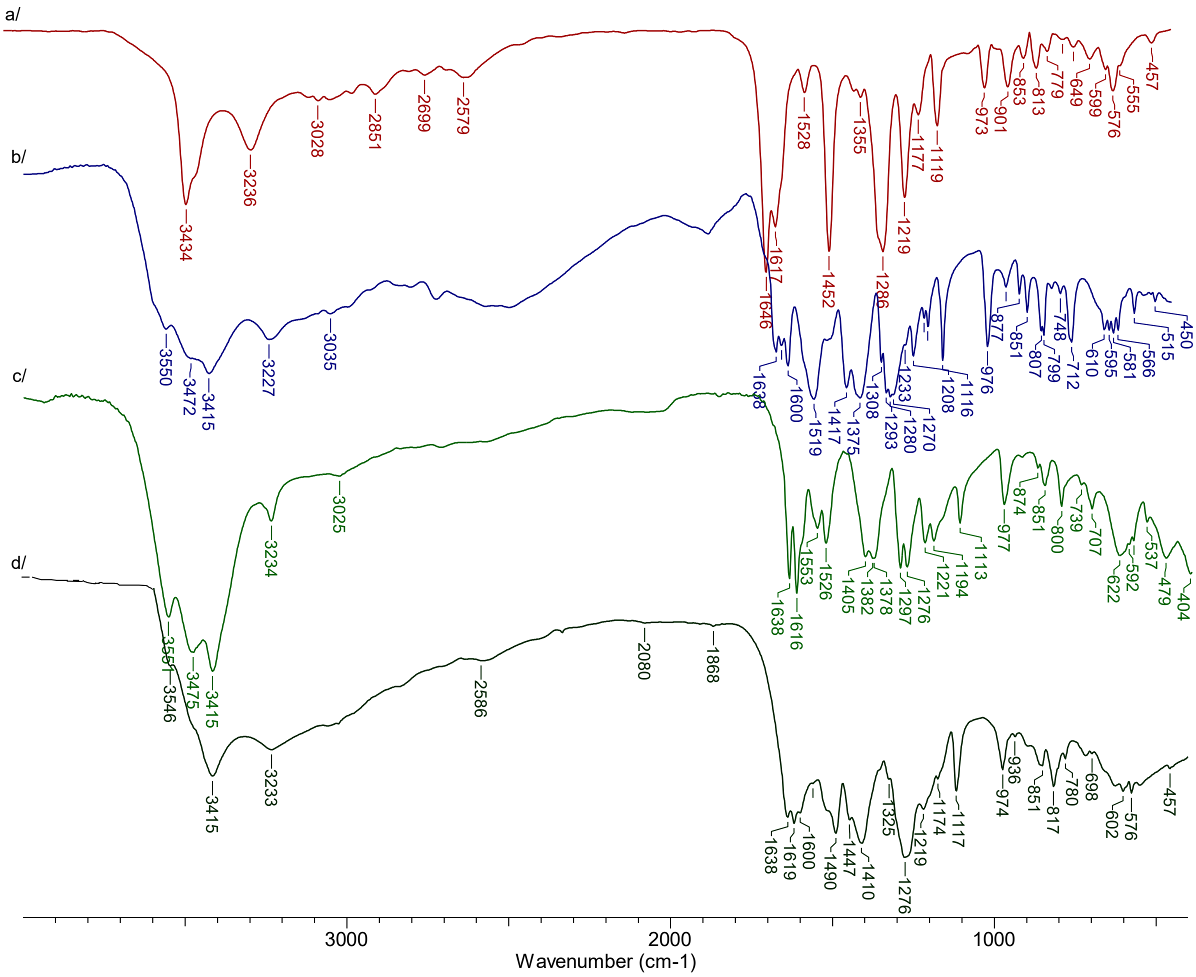

Figure S1. FT-IR spectra of CA (a), CA-Na (b), CA-K, CA-Cr(III) in the range of 4000–400 cm^{-1}.

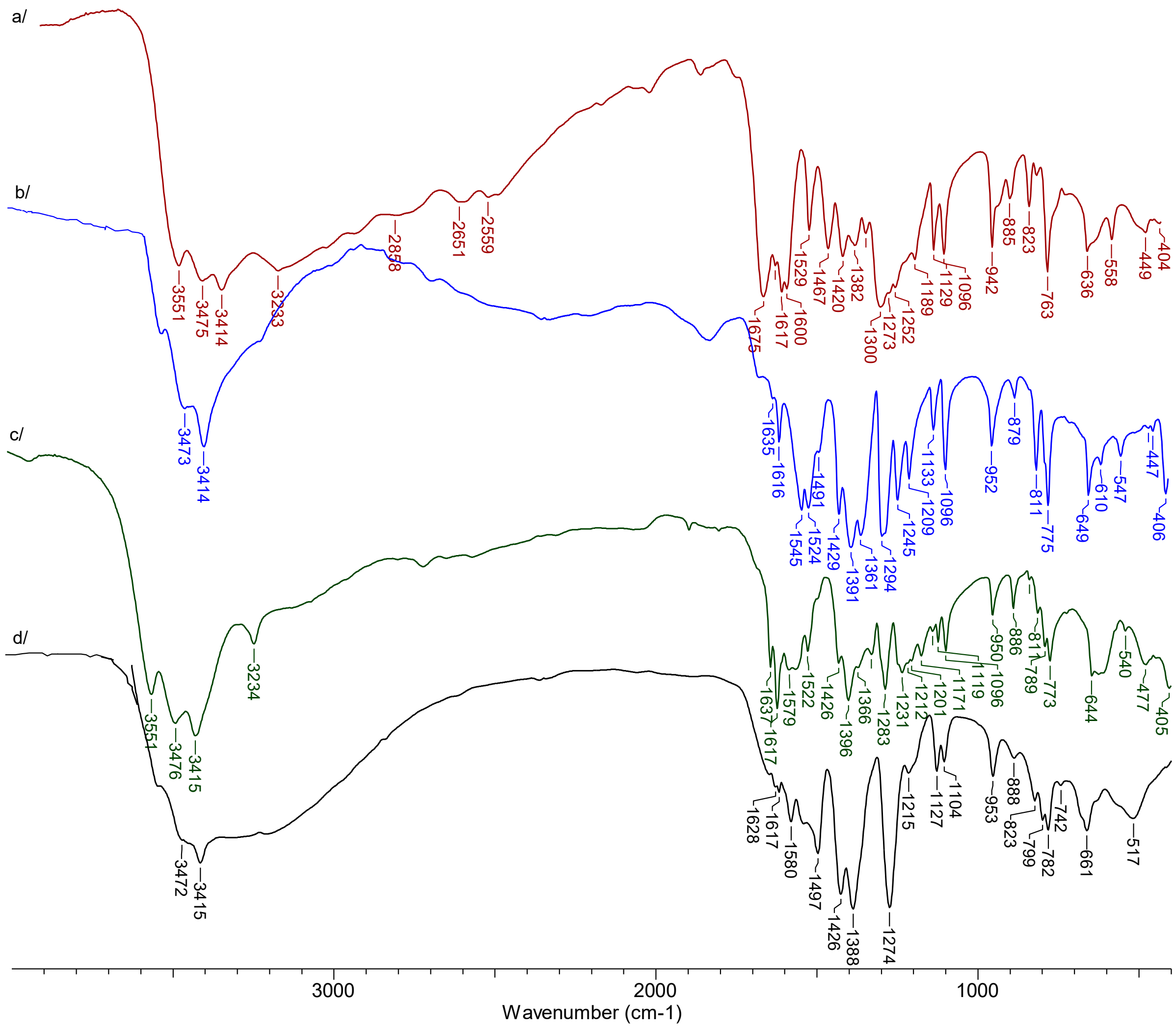

Figure S2. FT-IR spectra of 3,4-DHBA (a), 3,4-DHBA -Na (b), 3,4-DHBA -K, 3,4-DHBA -Cr(III) in the range of 4000–400 cm^{-1}.
